# Supplementary material for: Teaching programming using eduScrum methodology
Source: PeerJ Comput Sci. 2024 Jan 23;10:e1822. doi: 10.7717/peerj-cs.1822 (PMC10909224; doi:10.7717/peerj-cs.1822)
Supplement: Supplemental Information 2 — Almost everything is translated, except for 2-3 questions with an open answer, which were not used for statistical processing [file peerj-cs-10-1822-s002.pdf]

EduScrum

Milí respondenti, prosím o vyplnenie pravdivých informácií, nakoľko je tento dotazník súčasťou výskumu. Ďakujem.

Dear respondents, please fill in the truthful information as this questionnaire is part of the research. Thank you

* Povinné

1. Škola * — School - name

   Označte iba jednu elipsu.

   - ZŠ Ďumbierska - IX.A
   - ZŠ Ďumbierska - IX.B
   - Gymnázium Ľ. Štúra
   - Univerzita Mateja Bela v Banskej Bystrici
   - Gymnázium M. Rúfusa
   - Stredná odborná škola technická
   - Gymnázium Andreja Sládkoviča

2. Som * — Gender

   Označte iba jednu elipsu.

   - chlapec — boy
   - dievča — girl

3. Učil/a si sa formou eduScrum. Čo je podľa teba eduScrum? *

   You learned by eduScrum. What is eduScrum in your opinion?

4. Porovnaj bežné vyučovanie a eduScrum - k čomu sa viac prikloníš? *

   Compare regular teaching and eduScrum - which do you prefer?

   Označte iba jednu elipsu.

	1	2	3	4	5	
bežné vyučovanie						eduScrum

   classic teaching

5. Ako veľmi sa ti páčil eduScrum? *

   How much did you like eduScrum?

   Označte iba jednu elipsu.

	1	2	3	4	5	6	7	8	9	10	
vôbec sa mi nepáčil											super!

   I didn't like it at all

6. Pracoval si v tíme. Koľko vás bolo? *

you worked as part of a team. How many of you were there?

7. Na základe čoho ste utvorili tím? *

On what basis did you form the team?

8. Ako by si ohodnotil váš tím a spoluprácu? *

How would you rate your team and coop-eration?

Označte iba jednu elipsu.

	1	2	3	4	5	6	7	8	9	10	
zle sa nám spolupracovalo	⬭	⬭	⬭	⬭	⬭	⬭	⬭	⬭	⬭	⬭	spolupracovalo sa nám super

we had a bad cooper-ation

we had a great coop-eration

9. Čo si robil v tíme ty? *

What did you do in the team?

10. Myslíš, že sa s tebou dobre spolupracuje? Si tímový hráč? *

Do you think you're good to work with? Are you a team player?

Označte iba jednu elipsu.

- ⬭ áno
- ⬭ skôr áno
- ⬭ skôr nie
- ⬭ nie

yes

rather yes

rather no

no

11. Robil si na hodinách niečo kreatívne? Ak áno, čo to bolo?

Did you do anything creative in class? If yes, what was it?

12. Myslíš, že na eduScrum hodine môžeš byť viac kreatívny ako na bežnom vyučovaní? *

Do you think you can be more creative in an eduScrum class than in a regular class?

Označte iba jednu elipsu.

- áno
- skôr áno
- skôr nie
- nie
- Iné: _____

yes
rather yes
rather no
no
others:

13. Je dôležité plánovanie v eduScrum? *

Is task scheduling important in eduScrum?

Označte iba jednu elipsu.

- áno
- skôr áno
- skôr nie
- nie
- Iné: _____

yes
rather yes
rather no
no
others:

14. Je dôležité plánovanie úloh? (Nemáme na mysli len eduScrum, ale vo všeobecnosti) *

Task planning is important (I don't just mean eduScrum, but in general)

Označte iba jednu elipsu.

- áno
- skôr áno
- skôr nie
- nie
- Iné: _____

yes
rather yes
rather no
no
others:

15. "eduScrum prevráti vyučovanie hore nohami." Súhlasíš s týmto výrokom? *

eduScrum turns teaching upside down. Do you agree with this statement?

Označte iba jednu elipsu.

- áno
- skôr áno
- skôr nie
- nie
- Iné: _____

yes
rather yes
rather no
no
others:

16. Čo ti dali tieto vyučovacie hodiny? *

What did these lessons give you?

Začiarknite všetky vyhovujú[illegible]

yes rather yes rather no no

	áno	skôr áno	skôr nie	nie
fun	☐	☐	☐	☐
personal growth [illegible]iečo som sa naučil/a)	☐	☐	☐	☐
motivation for computer science [illegible]k [illegible]e	☐	☐	☐	☐
collaboration	☐	☐	☐	☐

17. Ako veľmi ti pomohol flipboard (plánovacia tabuľka)?

How much did the flipboard help you?

Označte iba jednu elipsu.

	0	1	2	3	4	5	
vôbec nepomohla	◯	◯	◯	◯	◯	◯	veľmi pomohla

did not help at all

helped a lot

18. Potreboval/a si často pomoc učiteľa? *

Have you often needed the help of a teacher?

Označte iba jednu elipsu.

- ◯ áno
- ◯ skôr áno
- ◯ skôr nie
- ◯ nie

yes
rather yes
rather no
no

19. Podstatou eduScrum-u je, že veľký problém sa dá vyriešiť vtedy, keď ho rozložíš na menšie problémy a riešiš problémy postupne. Súhlasíš s tým? *

The essence of eduScrum is that a big problem can be solved if you break it down into smaller problems and solve problems one at a time. Do you agree with that?

Označte iba jednu elipsu.

- ◯ áno
- ◯ skôr áno
- ◯ skôr nie
- ◯ nie

yes
rather yes
rather no
no

20. Ako sa ti páčil App Inventor? *

How did you like App Inventor?

Označte iba jednu elipsu.

	1	2	3	4	5	
vôbec nepáčil	◯	◯	◯	◯	◯	super

did not like at all

21. Ako si sa zlepšil v programovaní? *

How did you get better at programming?

Označte iba jednu elipsu.

	1	2	3	4	5	
vôbec som sa nezlepšil v programovaní	◯	◯	◯	◯	◯	dosť som sa zlepšil

I haven't improved my programming at all

I've improved quite a lot

22. Ako veľmi ťa bavili nasledujúce:

How much did you enjoy the following

V každom riadku označte iba jednu elipsu.

	vôbec nebavilo	trochu bavilo	veľmi bavilo	nerobili sme s nim
**App Inventor**	◯	◯	◯	◯
**Android Studio**	◯	◯	◯	◯
**micro:bit**	◯	◯	◯	◯
**MakeCode Arcade (programovanie 2D hry)**	◯	◯	◯	◯
**Minecraft: Educational Edition**	◯	◯	◯	◯
**Programovanie stránok (HTML CSS)**	◯	◯	◯	◯

Webpage programming

23. Čo si robil v App Inventore? *

If you did the App inventor - what did you do with it?

24. Ak si robil s nástrojom Android Studio - čo si s ním robil?

If you did Android Studio - what did you do with it?

25. Ak si robil s micro:bitom - čo si s ním robil?

If you did micro:bit - what did you do with it?

26. Ak si programoval hry v MakeCode Arcade - aké hry si v ňom robil?

If you've programmed games in MakeCode Arcade - what kind of games have you made in MakeCode Arcade?

27. Ak si robil s Minecraft: Education Edition - čo si v ňom robil?

If you've done Minecraft: Education Edition - what have you done in it?

28. Chcel by si sa aj naďalej učiť

Would you like to continue learning

V každom riadku ozn[illegible]

	yes	rather yes	rather no<br>nie	no	don't know/didn't do<br>som
**formou eduScrum?**	⬭	⬭	⬭	⬭	⬭
**v App Inventore?**	⬭	⬭	⬭	⬭	⬭
**v Android Studio?**	⬭	⬭	⬭	⬭	⬭
**s micro:bitom?**	⬭	⬭	⬭	⬭	⬭
**MakeCode Arcade (hry)**	⬭	⬭	⬭	⬭	⬭
**Minecraft**	⬭	⬭	⬭	⬭	⬭

29. Chcel by si niečo odkázať p. lektorovi?

Is there anything you would like to say to Mr. Lecturer?

Tento obsah nie je vytvorený ani schválený spoločnosťou Google.

Google Formuláre
