# Supplementary material for: Teaching programming using eduScrum methodology
Source: PeerJ Comput Sci. 2024 Jan 23;10:e1822. doi: 10.7717/peerj-cs.1822 (PMC10909224; doi:10.7717/peerj-cs.1822)
Supplement: Supplemental Information 3 [file peerj-cs-10-1822-s003.docx]

**Compare regular teaching and eduScrum - which do you prefer?**

Respondents could choose one of the following options: 1 is regular teaching and 5 is eduScrum. The results of the survey are shown in the table below.

H0: Teaching using eduScrum methodology is not significantly different for boys and girls.

H1: When teaching using eduScrum methodology, there are differences between the responses of boys and girls.

|  | Girls | Boys | Together |
| --- | --- | --- | --- |
| 1 (normal teaching) | 6 | 7 | 13 |
| 2 | 16 | 13 | 29 |
| 3 | 36 | 28 | 64 |
| 4 | 22 | 31 | 53 |
| 5 (eduScrum) | 12 | 27 | 39 |
| Together | 92 | 106 | 198 |
|  |  |  |  |
| Theoretical abundances: |  |  |  |
|  | Girls | Boys | Together |
| 1 (normal teaching) | 6,0 | 7,0 | 13,0 |
| 2 | 13,5 | 15,5 | 29,0 |
| 3 | 29,7 | 34,3 | 64,0 |
| 4 | 24,6 | 28,4 | 53,0 |
| 5 (eduScrum) | 18,1 | 20,9 | 39,0 |
| Spolu | 92,0 | 106,0 | 198,0 |
|  |  |  |  |
| probability of error: | | 10,18% |  |

We accept the null hypothesis.

H0: Teaching using the eduScrum methodology is not significantly different between primary and secondary school students.

H1: There are significant differences between the responses of primary and secondary school students when taught using eduScrum methodology.

|  | Primary schools | Secondary schools | Together |
| --- | --- | --- | --- |
| 1 (normal teaching) | 7 | 6 | 13 |
| 2 | 10 | 19 | 29 |
| 3 | 30 | 34 | 64 |
| 4 | 13 | 40 | 53 |
| 5 (eduScrum) | 8 | 31 | 39 |
| Spolu | 68 | 130 | 198 |
|  |  |  |  |
| Theoretical abundances:: |  |  |  |
|  | Primary schools | Secondary schools | Together |
| 1 (normal teaching) | 4,5 | 8,5 | 13,0 |
| 2 | 10,0 | 19,0 | 29,0 |
| 3 | 22,0 | 42,0 | 64,0 |
| 4 | 18,2 | 34,8 | 53,0 |
| 5 (eduScrum) | 13,4 | 25,6 | 39,0 |
| Spolu | 68,0 | 130,0 | 198,0 |
|  |  |  |  |
| probability of error: | | 1,58% |  |

We reject the null hypothesis.

How much did you like eduScrum?

1-10 by description

H0: There is no significant difference between boys and girls in the evaluation of eduScrum methodology.

Primary vs secondary schools

| Together | | 5731 | 13970 |  |  | 11766 | |
| --- | --- | --- | --- | --- | --- | --- | --- |
| number of objects n_1_ and n_2_: | | 68 | 130 |  |  |  |  |
| N | | 198 | |  |  |  |  |
| μ | | 4420 | |  |  |  |  |
| U | | 3385 | |  |  |  |  |
| σ_kor_ | | 379,38 | |  |  |  |  |
| u | | -2,728 | |  |  |  |  |
|  |  |  |  |  |  |  |  |
| **One-sided test** | |  |  | **Two-sided test** | |  |  |
| probability of error: | critical value |  |  | probability of error: | critical value |  |  |
| 5% | 1,64 |  |  | 5% | 1,96 |  |  |
| 1% | 2,33 |  |  | 1% | 2,58 |  |  |
| 0,10% | 3,09 |  |  | 0,10% | 3,29 |  |  |

**How would you rate your team and cooperation?**

| Together | | 9677,5 | 10023,5 |  |  | 36127,5 | |
| --- | --- | --- | --- | --- | --- | --- | --- |
| number of objects n_1_ and n_2_: | | 92 | 106 |  |  |  |  |
| N | | 198 | |  |  |  |  |
| μ | | 4876 | |  |  |  |  |
| U | | 5399,5 | |  |  |  |  |
| σ_kor_ | | 390,75 | |  |  |  |  |
| u | | 1,340 | |  |  |  |  |
|  |  |  |  |  |  |  |  |
| **One-sided test** | |  |  | **Two-sided test** | |  |  |
| probability of error: | critical value |  |  | probability of error: | critical value |  |  |
| 5% | 1,64 |  |  | 5% | 1,96 |  |  |
| 1% | 2,33 |  |  | 1% | 2,58 |  |  |
| 0,10% | 3,09 |  |  | 0,10% | 3,29 |  |  |

| Together | | 10418 | 14335 |  |  | 54742 | |
| --- | --- | --- | --- | --- | --- | --- | --- |
| počet objektov n_1_ a n_2_: | | 92 | 130 |  |  |  |  |
| N | | 222 | |  |  |  |  |
| μ | | 5980 | |  |  |  |  |
| U | | 6140 | |  |  |  |  |
| σ_kor_ | | 457,07 | |  |  |  |  |
| u | | 0,350 | |  |  |  |  |
|  |  |  |  |  |  |  |  |
| **One-sided test** | |  |  | **Two-sided test** | |  |  |
| probability of error: | critical value |  |  | probability of error: | critical value |  |  |
| 5% | 1,64 |  |  | 5% | 1,96 |  |  |
| 1% | 2,33 |  |  | 1% | 2,58 |  |  |
| 0,10% | 3,09 |  |  | 0,10% | 3,29 |  |  |

Primary vs secondary schools

| Together | | 10418 | 10446,5 |  |  | 31737 | |
| --- | --- | --- | --- | --- | --- | --- | --- |
| number of objects n_1_ and n_2_: | | 92 | 130 |  |  |  |  |
| N | | 222 | |  |  |  |  |
| μ | | 5980 | |  |  |  |  |
| U | | 6140 | |  |  |  |  |
| σ_kor_ | | 463,16 | |  |  |  |  |
| u | | 0,345 | |  |  |  |  |
|  |  |  |  |  |  |  |  |
| **One-sided test** | |  |  | **Two-sided test** | |  |  |
| probability of error: | critical value |  |  | probability of error: | critical value |  |  |
| 5% | 1,64 |  |  | 5% | 1,96 |  |  |
| 1% | 2,33 |  |  | 1% | 2,58 |  |  |
| 0,10% | 3,09 |  |  | 0,10% | 3,29 |  |  |
|  |  |  |  |  |  |  |  |
| If u is more than the critical value, we reject the null hypothesis. Otherwise, we accept it. | | | | | | | |

**Do you think you're good to work with? Are you a team player?**

H0: In the boys' and girls' responses to the question 'Do you think you are good to work with? Are you a team player?" there is no significant difference between the answers.

H1: There is no significant difference in the boys' and girls' responses to the question "Do you think you are good to work with? Are you a team player?" there is a difference between boys' and girls' responses.

|  |  |  |  |
| --- | --- | --- | --- |
|  | Girls | Boys | Together |
| yes | 46 | 47 | 93 |
| Rather yes | 40 | 49 | 89 |
| Rather no (+ no) | 6 | 10 | 16 |
| Together | 92 | 106 | 198 |
|  |  |  |  |
| Theoretical abundances: |  |  |  |
|  | Girls | Boys | Together |
| yes | 43,2 | 49,8 | 93,0 |
| Rather yes | 41,4 | 47,6 | 89,0 |
| Rather no (+ no) | 7,4 | 8,6 | 16,0 |
| Together | 92,0 | 106,0 | 198,0 |
|  |  |  |  |
| probability of error: | | 62,64% |  |

We accept the null hypothesis.

H0: In the responses of primary and secondary school students "Do you think you are good to work with? Are you a team player?" there is no significant difference between the responses.

H1: There is no significant difference in the responses of primary and secondary school students to the question "Do you think you are good to work with? Are you a team player?" there is a difference between the answers.

|  | Primary schools | Secondary schools | Together |
| --- | --- | --- | --- |
| yes | 32 | 63 | 95 |
| Rather yes | 28 | 58 | 86 |
| Rather no (+ no) | 8 | 9 | 17 |
| Together | 68 | 130 | 198 |
|  |  |  |  |
| Theoretical abundances: |  |  |  |
|  | Primary schools | Secondary schools | Together |
| yes | 32,6 | 62,4 | 95,0 |
| Rather yes | 29,5 | 56,5 | 86,0 |
| Rather no (+ no) | 5,8 | 11,2 | 17,0 |
| Together | 68,0 | 130,0 | 198,0 |
|  |  |  |  |
| probability of error: | | 50,69% |  |

We accept the null hypothesis.

**Do you think you can be more creative in an eduScrum class than in a regular class?**

H0: Boys' opinion on whether they are more creative during eduScrum lessons than in regular classes is not significantly different from girls' opinion.

H1: Boys' opinion on whether they are more creative in lessons using eduScrum methodology than in regular lessons is significantly different from girls' opinion.

|  | Girls | Boys | Together |
| --- | --- | --- | --- |
| yes | 40 | 45 | 85 |
| Rather yes | 46 | 54 | 100 |
| Rather no | 6 | 7 | 13 |
| Together | 92 | 106 | 198 |
|  |  |  |  |
| Theoretical abundances: |  |  |  |
|  | Girls | Boys | Together |
| yes | 39,5 | 45,5 | 85,0 |
| Rather yes | 46,5 | 53,5 | 100,0 |
| Rather no | 6,0 | 7,0 | 13,0 |
| Together | 92,0 | 106,0 | 198,0 |
|  |  |  |  |
| probability of error: | | 98,94% |  |

We accept the null hypothesis.

H0: The opinion of primary school students on whether they are more creative in the classroom during eduScrum than in the regular classroom is not significantly different from the opinion of secondary school students.

H1: The opinion of primary school pupils whether they are more creative in lessons during teaching using eduScrum methodology than in regular teaching is significantly different from the opinion of secondary school pupils.

|  | Primary schools | Secondary schools | Together |
| --- | --- | --- | --- |
| yes | 23 | 62 | 85 |
| Rather yes | 39 | 61 | 100 |
| Rather no | 6 | 7 | 13 |
| Together | 68 | 130 | 198 |
|  |  |  |  |
| Theoretical abundances: |  |  |  |
|  | Primary schools | Secondary schools | Together |
| yes | 29,2 | 55,8 | 85,0 |
| Rather yes | 34,3 | 65,7 | 100,0 |
| Rather no | 4,5 | 8,5 | 13,0 |
| Together | 68,0 | 130,0 | 198,0 |
|  |  |  |  |
| probability of error: | | 15,21% |  |

We accept the null hypothesis.

**Is planning important in eduScrum? Compared to the questions: is task scheduling important? (We don't mean just eduScrum, but in general)**

H0: There is no significant relationship between respondents' answers on the importance of scheduling in eduScrum and the importance of scheduling in general.

H1: There is a significant association between respondents' answers on the importance of planning in the eduScrum methodology and the importance of planning in general.

|  | Planning eduScrum | Planning in general | Together |
| --- | --- | --- | --- |
| yes | 95 | 112 | 207 |
| Rather yes | 82 | 75 | 157 |
| Rather no | 21 | 11 | 32 |
| Together | 198 | 198 | 396 |
|  |  |  |  |
| Theoretical abundances: |  |  |  |
|  | Planning eduScrum | Planning in general | Together |
| yes | 103,5 | 103,5 | 207,0 |
| Rather yes | 78,5 | 78,5 | 157,0 |
| Rather no | 16,0 | 16,0 | 32,0 |
| Together | 198,0 | 198,0 | 396,0 |
|  |  |  |  |
| probability of error: | | 8,92% |  |

We accept the null hypothesis.

**Have you often needed help from a teacher?**

H0: The differences in boys' and girls' responses to the question whether they needed help from a teacher are not significantly different.

H1: There are differences in boys' and girls' responses to the question whether they needed help from a teacher.

|  | Girls | Boys | Together |
| --- | --- | --- | --- |
| daily | 23 | 12 | 35 |
| at least once a week | 38 | 48 | 86 |
| at least once a month | 20 | 34 | 54 |
| less than once a month | 11 | 12 | 23 |
| Together | 92 | 106 | 198 |
|  |  |  |  |
| Theoretical abundances: |  |  |  |
|  | Dievčatá | Chlapci | Spolu |
| daily | 16,3 | 18,7 | 35,0 |
| at least once a week | 40,0 | 46,0 | 86,0 |
| at least once a month | 25,1 | 28,9 | 54,0 |
| less than once a month | 10,7 | 12,3 | 23,0 |
| Together | 92,0 | 106,0 | 198,0 |
|  |  |  |  |
| probability of error: | | 6,18% |  |

We accept the null hypothesis.

H0: The differences in responses between primary and secondary school students on whether they needed help from a teacher are not significantly different.

H1: There are significant differences in the responses of primary and secondary school pupils to the question whether they needed help from a teacher.

|  | Primary schools | Secondary schools | Together |
| --- | --- | --- | --- |
| daily | 15 | 20 | 35 |
| at least once a week | 35 | 51 | 86 |
| less than once a week | 18 | 59 | 77 |
| Together | 68 | 130 | 198 |
|  |  |  |  |
| Theoretical abundances: |  |  |  |
|  | Primary schools | Secondary schools | Together |
| daily | 12,0 | 23,0 | 35,0 |
| at least once a week | 29,5 | 56,5 | 86,0 |
| less than once a week | 26,4 | 50,6 | 77,0 |
| Together | 68,0 | 130,0 | 198,0 |
|  |  |  |  |
| probability of error: | | 3,38% |  |

We reject the null hypothesis.

**The essence of eduScrum is that a big problem can be solved if you break it down into smaller problems and solve problems incrementally. Do you agree with that?**

H0: There are no significant differences in boys' and girls' responses to the question 'The essence of eduScrum is that a big problem can be solved when you break it down into smaller problems and solve problems one at a time. Do you agree with that?"

H1: Boys and girls answered differently to the question that the essence of eduScrum is that a big problem can be solved if you break it down into smaller problems one at a time.

|  | Girls | Boys | Together |
| --- | --- | --- | --- |
| yes | 40 | 51 | 91 |
| Rather yes | 44 | 44 | 88 |
| Rather no, no | 8 | 11 | 19 |
| Together | 92 | 106 | 198 |
|  |  |  |  |
| Theoretical abundances: |  |  |  |
|  | Dievčatá | Chlapci | Spolu |
| yes | 42,3 | 48,7 | 91,0 |
| Rather yes | 40,9 | 47,1 | 88,0 |
| Rather no, no | 8,8 | 10,2 | 19,0 |
| Together | 92,0 | 106,0 | 198,0 |
|  |  |  |  |
| probability of error: | | 66,45% |  |

H0: There are significant differences in the responses of primary and secondary school students to the question "The essence of eduScrum is that a big problem can be solved if you break it down into smaller problems and solve problems one at a time. Do you agree with that?"

H1: Primary and secondary school students answered differently to the question that the essence of eduScrum is that a big problem can be solved if you break it down into smaller problems one at a time.

|  | Primary schools | Secondary schools | Together |
| --- | --- | --- | --- |
| yes | 25 | 66 | 91 |
| Rather yes | 36 | 52 | 88 |
| Rather no, no | 7 | 12 | 19 |
| Together | 68 | 130 | 198 |
|  |  |  |  |
| Theoretical abundances: |  |  |  |
|  | Primary schools | Secondary schools | Together |
| yes | 31,3 | 59,7 | 91,0 |
| Rather yes | 30,2 | 57,8 | 88,0 |
| Rather no, no | 6,5 | 12,5 | 19,0 |
| Together | 68,0 | 130,0 | 198,0 |
|  |  |  |  |
| probability of error: | | 16,20% |  |

**Ako sa ti páčil App Inventor?**

H_0_: Medzi názorom chlapcov a dievčat, či sa im nástroj App Inventor páčil nie sú štatisticky významné rozdiely.

H1: Existujú rozdiely medzi názorom chlapcov a dievčat, či sa im nástroj App Inventor páčil.

|  | Girls | Boys | Together |
| --- | --- | --- | --- |
| 1 (v) | 7 | 9 | 16 |
| 2 | 12 | 11 | 23 |
| 3 | 24 | 23 | 47 |
| 4 | 21 | 27 | 48 |
| 5 (super) | 28 | 36 | 64 |
| Together | 92 | 106 | 198 |
|  |  |  |  |
| Theoretical abundances: |  |  |  |
|  | Dievčatá | Chlapci | Spolu |
| 1 (did not like at all) | 7,4 | 8,6 | 16,0 |
| 2 | 10,7 | 12,3 | 23,0 |
| 3 | 21,8 | 25,2 | 47,0 |
| 4 | 22,3 | 25,7 | 48,0 |
| 5 (super) | 29,7 | 34,3 | 64,0 |
| Together | 92,0 | 106,0 | 198,0 |
|  |  |  |  |
| probability of error: | | 89,74% |  |

We accept the null hypothesis.

H0: There are no statistically significant differences between primary and secondary school students' opinions on whether they liked App Inventor.

H1: There are differences between the opinion of primary and secondary school students whether they liked the App Inventor tool.

|  | Primary schools | Secondary schools | Together |
| --- | --- | --- | --- |
| 1 (did not like at all) | 10 | 6 | 16 |
| 2 | 14 | 9 | 23 |
| 3 | 24 | 23 | 47 |
| 4 | 8 | 40 | 48 |
| 5 (super) | 12 | 52 | 64 |
| Together | 68 | 130 | 198 |
|  |  |  |  |
| Theoretical abundances: |  |  |  |
|  | Primary schools | Secondary schools | Together |
| 1 (did not like at all) | 5,5 | 10,5 | 16,0 |
| 2 | 7,9 | 15,1 | 23,0 |
| 3 | 16,1 | 30,9 | 47,0 |
| 4 | 16,5 | 31,5 | 48,0 |
| 5 (super) | 22,0 | 42,0 | 64,0 |
| Together | 68,0 | 130,0 | 198,0 |
|  |  |  |  |
| probability of error: | | 0,00% |  |

We reject the null hypothesis.

How did you improve in programming?

H0: Among boys and girls, when asked "How have you improved in programming?" there are no statistically significant differences.

H1: There are no significant differences between boys and girls on the question "How did they improve in programming?" there are differences.

|  | Girls | Boys | Together |
| --- | --- | --- | --- |
| 1 a 2(ever) | 24 | 14 | 38 |
| 3 | 36 | 33 | 69 |
| 4 | 24 | 36 | 60 |
| 5 | 8 | 23 | 31 |
| Together | 92 | 106 | 198 |
|  |  |  |  |
| Theoretical abundances: |  |  |  |
|  | Girls | Boys | Together |
| 1 a 2(ever) | 17,7 | 20,3 | 38,0 |
| 3 | 32,1 | 36,9 | 69,0 |
| 4 | 27,9 | 32,1 | 60,0 |
| 5 | 14,4 | 16,6 | 31,0 |
| Together | 92,0 | 106,0 | 198,0 |
|  |  |  |  |
| probability of error: | | 0,94% |  |

We reject the null hypothesis.

H0: Among elementary and middle school students, when asked "How have they improved in programming?" there are no statistically significant differences.

H1: There are no significant statistical differences between primary and secondary school pupils on the question "How did they improve in programming?" there are differences.

|  | Primary schools | Secondary schools | Together |
| --- | --- | --- | --- |
| 1 a 2(ever) | 23 | 15 | 38 |
| 3 | 25 | 44 | 69 |
| 4 | 15 | 45 | 60 |
| 5 | 5 | 26 | 31 |
| Together | 68 | 130 | 198 |
|  |  |  |  |
| Theoretical abundances: |  |  |  |
|  | Primary schools | Secondary schools | Together |
| 1 a 2(ever) | 13,1 | 24,9 | 38,0 |
| 3 | 23,7 | 45,3 | 69,0 |
| 4 | 20,6 | 39,4 | 60,0 |
| 5 | 10,6 | 20,4 | 31,0 |
| Together | 68,0 | 130,0 | 198,0 |
|  |  |  |  |
| probability of error: | | 0,03% |  |

We reject the null hypothesis.

Would you like to continue learning with the eduScrum methodology?

H0: There are no significant differences in the responses of boys and girls as to whether they would like to continue learning using the eduScrum methodology.

H1: There are significant differences in boys' and girls' responses as to whether they would like to continue teaching using the eduScrum methodology.

|  |  |  |  |
| --- | --- | --- | --- |
|  | Girls | Boys | Together |
| yes | 21 | 32 | 53 |
| rather yes | 46 | 49 | 95 |
| rather no | 16 | 17 | 33 |
| No | 9 | 8 | 17 |
| together | 92 | 106 | 198 |
|  |  |  |  |
| Theoretical abundances: |  |  |  |
|  | dievčatá | chlapci | Spolu |
| yes | 24,6 | 28,4 | 53,0 |
| rather yes | 44,1 | 50,9 | 95,0 |
| rather no | 15,3 | 17,7 | 33,0 |
| No | 7,9 | 9,1 | 17,0 |
| together | 92,0 | 106,0 | 198,0 |
|  |  |  |  |
| probability of error: | | 68,59% |  |

We accept the null hypothesis.

H0: There are no significant differences in the responses of primary and secondary school students on whether they would like to continue learning using the eduScrum methodology.

H1: There are significant differences in the responses of pupils from primary and secondary schools on whether they would like to continue teaching using the eduScrum methodology.

|  |  |  |  |
| --- | --- | --- | --- |
|  | Primary schools | Secondary schools | Together |
| yes | 13 | 40 | 53 |
| rather yes | 34 | 61 | 95 |
| rather no | 12 | 21 | 33 |
| No | 9 | 8 | 17 |
| together | 68 | 130 | 198 |
|  |  |  |  |
| Theoretical abundances: |  |  |  |
|  | Primary schools | Secondary schools | Together |
| yes | 18,2 | 34,8 | 53,0 |
| rather yes | 32,6 | 62,4 | 95,0 |
| rather no | 11,3 | 21,7 | 33,0 |
| No | 5,8 | 11,2 | 17,0 |
| together | 68,0 | 130,0 | 198,0 |
|  |  |  |  |
| probability of error: | | 17,04% |  |

We accept the null hypothesis.

In App Inventor

H0: There are no significant differences in the responses of boys and girls on whether they would like to continue learning mobile app programming in App Inventor.

H1: There are significant differences in the responses of boys and girls whether they would like to continue learning mobile app programming in App Inventor.

|  | Girls | Boys | Together |
| --- | --- | --- | --- |
| yes | 22 | 37 | 59 |
| rather yes | 42 | 40 | 82 |
| rather no | 19 | 16 | 35 |
| No | 9 | 13 | 22 |
| together | 92 | 106 | 198 |
|  |  |  |  |
| Theoretical abundances: |  |  |  |
|  | dievčatá | chlapci | Spolu |
| yes | 27,4 | 31,6 | 59,0 |
| rather yes | 38,1 | 43,9 | 82,0 |
| rather no | 16,3 | 18,7 | 35,0 |
| No | 10,2 | 11,8 | 22,0 |
| together | 92,0 | 106,0 | 198,0 |
|  |  |  |  |
| probability of error: | | 27,51% |  |

We accept the null hypothesis.

H0: There are no significant differences in the responses of primary and secondary school students on whether they would like to continue learning mobile app programming in App Inventor.

H1: There are significant differences in the responses of students from primary and secondary schools on whether they would like to continue learning mobile app programming in App Inventor.

|  | Primary schools | Secondary schools | Together |
| --- | --- | --- | --- |
| yes | 15 | 44 | 59 |
| rather yes | 24 | 58 | 82 |
| rather no | 17 | 18 | 35 |
| No | 12 | 10 | 22 |
| together | 68 | 130 | 198 |
|  |  |  |  |
| Theoretical abundances: |  |  |  |
|  | Primary schools | Secondary schools | Together |
| yes | 20,3 | 38,7 | 59,0 |
| rather yes | 28,2 | 53,8 | 82,0 |
| rather no | 12,0 | 23,0 | 35,0 |
| No | 7,6 | 14,4 | 22,0 |
| together | 68,0 | 130,0 | 198,0 |
|  |  |  |  |
| probability of error: | | 1,74% |  |
